# Isolation and Phenotyping of Normal Mouse Liver Dendritic Cells by an Improved Method

**Published:** 2011

**Authors:** Ghasem Mosayebi, Sayyed Mohammad Moazzeni

**Affiliations:** 1 * Molecular and Medicine Research Center, School of Medicine, Arak University of Medical Sciences, Arak, Iran*; 2 *Department of Immunology, School of Medical Sciences, Tarbiat Modarres University, Tehran, Iran*

**Keywords:** Dendritic cells, Liver, Mice, Phenotype

## Abstract

**Introduction:**

Dendritic cells (DCs) are bone marrow-derived cells, which migrate to lymphoid and non-lymphoid organs via blood. Liver DCs are believed to play an important role in the regulation of hepatic allograft acceptance. However, because of inherent difficulties in isolating adequate numbers of DCs from liver, limited information is available on the phenotype and functions of liver DCs. To address this issue, we isolated DCs from normal C57BL/6 mouse liver using a modified procedure and described their immunophenotypic characteristics.

**Materials and Methods:**

Non-parenchymal cells (NPCs) were obtained by collagenase digestion of perfused liver fragments and density gradient centrifugation (14.5% nycodenz column). After overnight (18 hr) incubation of the NPCs, enrichment for transiently adherent, low- density cells on 13% nycodenz gradients permitted the recovery of low numbers of cells (approximately 1.2-1.5 x 10^5^ per liver), many of which displayed distinct DCs morphology (abundant cytoplasm with prominent projections and irregularly shaped nuclei).

**Results:**

Flowcytometric analysis revealed that most of these cells were recognized by anti-CD11c (60-70%). The results obtained from double staining with PE and FITC conjugated monoclonal antibodies indicated that these cells were CD11c^+^/MHC-II^+^ (53%), CD11c^+^/CD86^+ ^(53.5%), CD11c^+^/ CD8α^+^ (36%) and CD11c^+^/CD11b^+ ^(45%).

**Conclusion:**

These findings indicate that the purity of DCs isolated by nycodenz gradient is higher than other reported methods. Considering the similar ratio of lymphoid (CD11c^+^/CD8α^+^) and myeloid (CD11c^+^/CD11b^+^) DCs in the liver, and the known role of lymphoid DCs in tolerance induction, it seems that this subpopulation of DCs is not the main reason of liver tolerogenecity. Therefore, other factors such as the immaturity of liver DCs or the effect of liver microenvironment on these cells, etc. may explain the acceptance of hepatic allograft.

## Introduction

Dendritic cells (DCs) are distributed ubiquitously throughout the body and play an important role in inducing immunity and/or tolerance (1,2). Several DCs subgroups with different biological roles have been identified (3,4). Myeloid DCs usually induce immune response and tolerance and usually are induced by lymphoid DCs (5,6). Difference in frequency and ratio of DCs subtypes appears to be one of the reasons for the differences in immune response in various tissues (7). Donor liver dendritic cells have been identified within various lymphoid and non-lymphoid tissues of organ allograft recipients, including immunosuppressor non-treated mice transplanted with and permanently accepting major histocompatibility complex (MHC)- incompatible hepatic allograft (8-10). Freshly isolated DCs from lymphoid organs are potent stimulators of primary MLR (11). Whereas, freshly isolated DCs of liver are unable to initiate primary T cell responses (12). Therefore, DCs may play an important role in modulation of the immunological responses and tolerance induction in liver transplantation (13). It is thought that the DCs resident within non-lymphoid tissues may be functionally immature and/or more heterogeneous than the potent stimulators of T cell activation that can be isolated from lymphoid organs (14,15). However, several questions have risen about the basis of the tolerogenicity of the liver, in particular, about the phenotype of liver DCs.

The phenotype and properties of liver DCs have been described both in the rat and man (16,17). However, because of inherent difficulties in isolating adequate number of mouse liver DCs, there are few studies on phenotype and function of mouse liver DCs (11,18). Furthermore, there are some reports regarding the properties of liver-derived DCs from GM-CSF or Flt3-L treated mice (19,20), but little is known about the properties and phenotype of DCs present within normal mouse liver. However, DCs isolated from liver mouse in presence of GM-CSF and Flt3-L or DCs derived from CD34^+ ^progenitor cells may affect phenotype and function of DCs. 

Since liver is the most tolerogenic of all transplanted organs and its graft can be accepted across MHC barriers in mice without the need for immunosuppressive therapy (21), studies on mouse liver DCs may provide important clues to understand mechanisms underlying tolerance induction. In this study, we have isolated DC-enriched cell populations from normal mouse liver with a modified separation method and described their immunophenotype.

## Materials and Methods

***Mice***

Adult 6-8 weeks old female C57BL/6 mice were purchased from the Razi vaccine and serum research . 

***Isolation of NPCs from liver***

**Figure 1.**
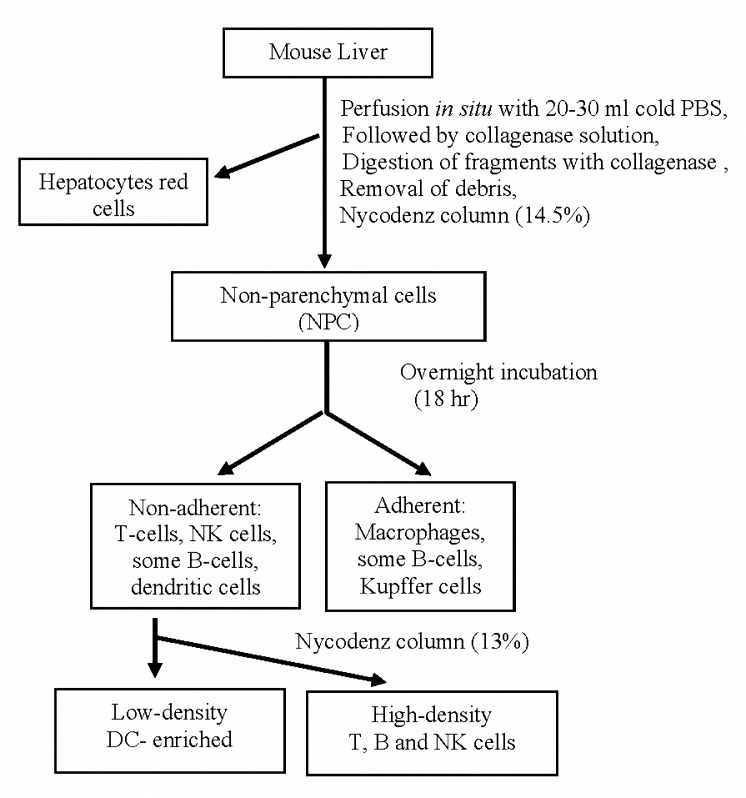
Flow plan for the isolation of non-parenchymal and DCs-enriched cells from normal mouse liver

Mice were anesthetized with ketamine, swabbed with 70% ethanol and an abdominal mid-line incision was performed. NPCs were isolated from the liver of mice, as Rao *et al* have described previously (22), with the following modifications (Figure 1). Briefly, the liver was perfused for 3 min *in situ* via the inferior vena cava, using 20-30 ml ice-cold phosphate buffer saline (PBS) and a 22 G intravenous catheter. 2 ml collagenase solution (Roche, Mannheim, Germany; 1mg/ml) in RPMI-1640 (Gibco, BRL) was then injected. The liver was excised immediately, diced into small pieces and digested in collagenase solution (5 ml/liver) for 30 min at 37 °C, with constant stirring. The digested tissue was then filtered through a 0.1 mm sterile wire mesh. Cells from three to four livers were pooled. The cell suspension was washed twice with PBS or RPMI-1640 medium by centrifugation at 400 g for 5 min at 4 °C. The final pellet was re-suspended in 2-3 ml RPMI medium and overlaid on nycodenz ( , ) gradient 14.5% (w/v) and centrifuged at 600 g for 15 min at 4 °C. The recovered low density (LD) cells from the interface were collected using a Pasteur pipet and washed as mentioned above.

***Enrichment of dendritic cells***

To enrich the DCs, NPCs were suspended in complete tissue culture medium (RPMI-1640 containing 10% FCS) and incubated overnight (18 hr) at 37 **°**C, 5% CO_2_ in tissue culture petridishes. At the end of incubation time, non-adherent cells were recovered and layered on a 3 ml column of 13% (w/v) nycodenz and centrifuged at 600 g for 15 min at 4** °**C. Low-density cells separated carefully from interface using a Pasteur pipette. These cells referred to as “DCs-enriched cells”, were washed and used for flowcytometric analysis.

***Flow cytometric analysis***

DC-enriched cells (1×10^5^ cells/tube) in flow cytometric buffer (PBS with 2% FCS and 0.01% sodium azide; Sigma) were stained by indirect double staining immunofluorescence technique. Briefly, the cells surface was first blocked with 5% normal mouse serum for 10 min on ice and then stained with primary monoclonal antibodies (mAbs): hamster anti-CD11c (clone HL3), rat anti-MHC class II (clone 2G9), rat anti-CD8α (clone 53-6.7), rat ant-CD11b (clone M1/70), and rat anti-CD86 (clone GL1). All of mAbs were purchased from PharMingen Company. Optimal concentration of hamster anti-mouse CD11c was added to all tubes (except the tube of isotype control) and then rat anti-mouse CD86, MHC-II, CD11b and CD8α were added to each tube, separately. The species and isotype-matched irrelevant antibodies were used as negative controls. The cells were then incubated on ice for 30 min and washed with flow cytomertic buffer. Phycoerythrin (PE)- conjugated mouse anti-hamster IgG (clone G70-204) and fluoresein isothiocyanate, (FITC)-conjugated mouse anti-rat IgG2a (clone RG7/1.30), and IgG2b (clone RG7/11.1) were used as secondary antibodies. After staining, the cells were fixed in 1% paraformaldehyde and flow cytometric

analysis was performed using FACSscan (Culter). 

## Results

***Isolation of hepatic NPCs***

**Figure 2.**
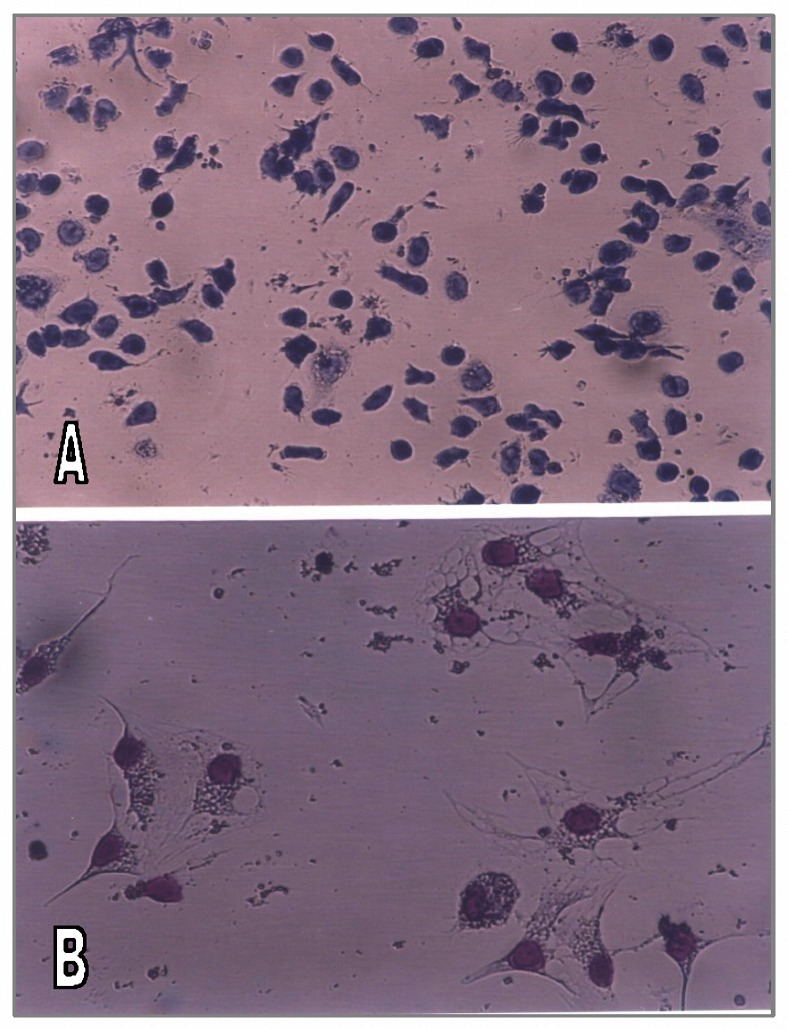
Giemsa-stained cytocentrifuge preparations of freshly isolated Non-parenchymal cells (A) and low-density DC enriched cells recovered from nycodenz gradients after overnight culture of NPCs from normal C57BL/6 mouse liver (B). The cells are agranular, with variable degrees of cytoplasmic vacuolation and irregularly shaped distinct cytoplasmic projections.

Liver tissues were digested with collagenase and NPCs were separated on a 14.5% nycodenz column. The number of NPCs isolated from a normal mouse liver was approximately 4x10^6 ^cells, with less than 5% hepatocyte contamination on microscopic examination (Figure 2A). DC population (CD11c^+^ cells) constituted ≤ 1.0% of the normal nonparenchymal cells.

To enrich the DCs, freshly isolated NPCs were cultured overnight at 37 °C and 5% CO_2_. Non-adherent cells were recovered and layered on a nycodenz column (13% w/v). Low-density fraction was separated and referred to as DC-enriched cells. DC population was enriched 20-30% after overnight culture and 13% nycodenz gradient centrifugation. 1.2-1.5 ×10^5 ^DC-enriched cells were isolated from each mouse liver. This population consisted of mononuclear cells with distinct DC morphology. Giemsa-stained preparations exhibited cells with irregularly shaped eccentric nuclei and abundant cytoplasmic projections or veils (Figure 2B). Double indirect immunostaining was used to detect co-expression of MHC class-II, CD86, CD8α and CD11b molecules with CD11c on enriched cell populations. The harvested low-density cell fraction was incubated with primary monoclonal antibodies then stained by PE and FITC- conjugated monoclonal antibodies. 

**Figure 3.**
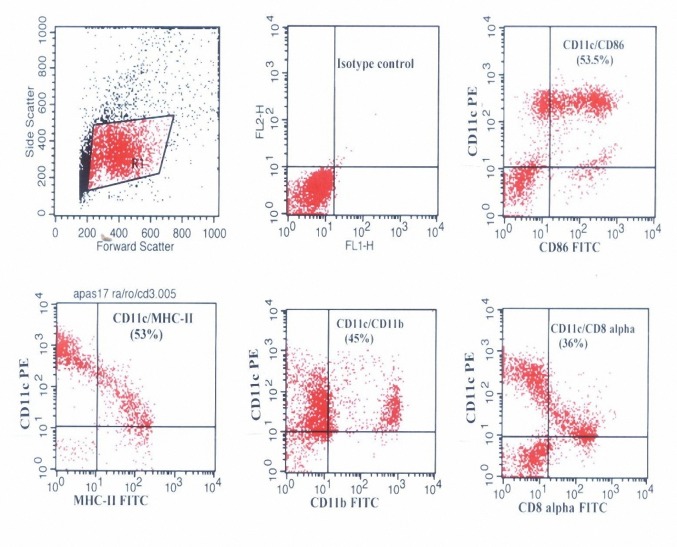
Flow cytometric analysis of the cell surface markers on the DC-enriched cells. Non-adherent LD cells were harvested from nycodenz gradients after overnight culture of NPCs from normal C57BL/6 mouse liver. The cells were stained by indirect immunofluorescence. The results are representative of 5 separate experiments.

The obtained results showed that, approximately 65% of the DC-enriched cells possess CD11c marker. The levels of MHC-II and co stimulatory molecule (CD86) expression on CD11c positive cells were 53% and 53.5%, respectively. 45% and 36% of CD11c^+^-cells express the myeloid marker (CD11b) and lymphoid marker (CD8α) respectively (Figure 3).

## Discussion

Dendritic cells (DCs) are a minor population of bone marrow-derived leukocytes that are distributed ubiquitously throughout the body (23). DCs are resident in the interstitial connective tissue of non-lymphoid organs, such as the liver, kidney or heart. They are believed to be important passenger leukocytes that migrate to T dependent areas of host lymphoid tissue following organ transplantation (24). The participation of liver DCs in development of tolerance to grafted tissue has been suggested in previous studies (13). It has been suggested that the chimeric cells, which are observed in various organs of recipients of liver or other allografts consist predominantly of DC lineage cells (21,25). However, the study of DCs located in the liver has been restricted by their relative scarcity and difficulty of their isolation and consequently limited information is available on characteristics of these cells. Some studies have showed that injection of Flt3-Ligand to mouse or culture of DCs in the presence of GM-CSF increases the number of liver DCs dramatically (19,26). However, these cytokines probably affect the function and/or phenotype of DCs. In the present study, we used a modified method (Nycodenz gradient) for isolating of DCs from normal mouse liver without using cytokines or coarse manipulation. We isolated 4x10^6 ^non-parenchymal cells from each liver. After overnight-culture of NPCs and separating low-density cells by nycodenz gradient, approximately 1.2-1.5x10^5^ cells/liver had DC-like morphology. This degree of enrichment is similar to that reported by others (11, 27,28). Following flow cytometric analysis, it was found that the great majority (60-70%) of these cells possess DC-related marker (CD11c^+^). 

Some previous studies have suggested that freshly-isolated DCs from non-lymphoid tissues, such as liver, are functionally immature and are more heterogeneous than the lymphoid organs (15,29). In contrast, the results of this study showed that the majority of isolated DCs possess phenotype of mature DCs with high expression of MHC class-II and co stimulatory molecule, CD86 on their surface. Abe *et al* have reported that the expression of MHC class-II and CD86 on GM-CSF treated liver derived DCs were 30% and 20%, respectively (28). Other investigators showed that DCs isolated from mice treated with Flt3-L are phenotypically immature (CD11c^+^, CD86 ^Low^, MHC-II ^Low)^ (26). However, the phenotypic changes after overnight culture have also been reported for DCs isolated from other mouse non-lymphoid organs (27). Therefore, some of these variations are probably because of culturing.

Freshly isolated DCs from lymphoid organs are potent stimulators of primary MLR, whereas freshly isolated DCs from non-lymphoid organs are unable to initiate primary T cell responses (30,31). These findings may reflect the heterogeneity of DC populations residing in different organs (32). The CD11c^+^ DC population can be divided into at least two subsets on the basis of the expression of CD11b and CD8α, which may represent the putative myeloid and lymphoid related subsets (33). Our results show that the frequency of DCs in freshly isolated normal liver NPCs is very low. However, CD8α^+^ and CD11b^+^ DCs were substantially enriched to 36 and 45 percent respectively by overnight culture and nycodenz density centrifugation. The obtained results also indicate that both subsets were present in normal mouse liver and in approximately equivalent numbers. O’Connel *et al* also showed that the frequency of lymphoid and myeloid DC subset is equal in the liver (27). However, we could not find an obvious difference between DCs isolated from the liver and the spleen (not shown data) of normal mouse.

## Conclusions

These findings indicate that purity of isolated DCs using nycodez gradient is higher than other reports (34,35). The similar ratio of lymphoid (CD11c^+^/CD8α^+^) and myeloid (CD11c^+^/CD11b^+^) DCs in liver demonstrated that the difference in frequency of these subpopulations of dendritic cells is not the main reason for the tolerance to liver transplants. However hepatic microenvironment may play an important role in tolerogenicity of DCs in this organ.
